# Structural Stability Monitoring of a Physical Model Test on an Underground Cavern Group during Deep Excavations Using FBG Sensors

**DOI:** 10.3390/s150921696

**Published:** 2015-08-31

**Authors:** Yong Li, Hanpeng Wang, Weishen Zhu, Shucai Li, Jian Liu

**Affiliations:** 1 Geotechnical & Structural Engineering Research Center, Shandong University, Jinan 250061, China; E-Mails: yongli@sdu.edu.cn (Y.L.); zhuw@sdu.edu.cn (W.Z.); lishucai@sdu.edu.cn (S.L.); 2 School of Civil Engineering, Shandong University, Jinan 250061, China; E-Mail: lj75@sdu.edu.cn

**Keywords:** structural stability monitoring, FBG sensor, physical model test, underground cavern group, numerical simulation

## Abstract

Fiber Bragg Grating (FBG) sensors are comprehensively recognized as a structural stability monitoring device for all kinds of geo-materials by either embedding into or bonding onto the structural entities. The physical model in geotechnical engineering, which could accurately simulate the construction processes and the effects on the stability of underground caverns on the basis of satisfying the similarity principles, is an actual physical entity. Using a physical model test of underground caverns in Shuangjiangkou Hydropower Station, FBG sensors were used to determine how to model the small displacements of some key monitoring points in the large-scale physical model during excavation. In the process of building the test specimen, it is most successful to embed FBG sensors in the physical model through making an opening and adding some quick-set silicon. The experimental results show that the FBG sensor has higher measuring accuracy than other conventional sensors like electrical resistance strain gages and extensometers. The experimental results are also in good agreement with the numerical simulation results. In conclusion, FBG sensors could effectively measure small displacements of monitoring points in the whole process of the physical model test. The experimental results reveal the deformation and failure characteristics of the surrounding rock mass and make some guidance for the *in situ* engineering construction.

## 1. Introduction

Currently, in the southwestern areas of China, numerous large-scale hydropower stations are being built, such as Ertan [1], Jinping I [2], Xiluodu [3], Dagangshan [4], Houziyan [5], and Baihetan [6]. Additionally, most of these sites require building an underground cavern group. Therefore, a large number of scientific problems urgently need to be solved regarding the stability of the underground group in the general processes of design and construction. Physical model tests, numerical modeling, and *in situ* monitoring are still three effective methods for investigating the stability of the underground cavern group. Physical model tests in geotechnical engineering could accurately simulate the excavation processes and effects on the stability of underground caverns on the basis of satisfying the similarity principles. The most important role of the physical model testing is to determine the internal stress and displacement field expected during cavern excavation. Ideally, the internal displacement results will reflect the related problems on the overall structural stability of the underground cavern group.

In the structural stability monitoring of the underground cavern group, a number of advanced measuring techniques have been utilized, such as electrical resistance strain gauges [7], Linear Variable Differential Transformers (LVDT) [8], mini multi-point extensometers [9], digital speckle photography deformation measurement (DSPD) [10], Fiber Bragg Grating (FBG) sensors [11,12,13], and others. For strain gauges bonding to the specimen for structural health monitoring, the resistive type strain gauges are always sensitive to temperature variation; therefore, it becomes necessary to account for variations in strain gauge resistance due to temperature changes. Additionally, some monitoring techniques do not respond well to instantaneous deformation associated with the cavern excavations. The main disadvantages of LVDTs are: (1) very high displacement is required for generating high voltages; (2) shielding is required since it is sensitive to magnetic field; (3) the performance of the transducer gets affected by vibrations; and (4) it is also greatly affected by temperature changes [14]. Although the multi-point extensometer has very high measuring accuracy, it is difficult to embed it into the physical model [14]. The DSPD measuring method is also utilized in the monitoring of larger displacement fields [14].

## 2. Principle and Design of the FBG Sensor in Displacement Monitoring

### 2.1. Principle of the FBG Sensor

**Figure 1:**
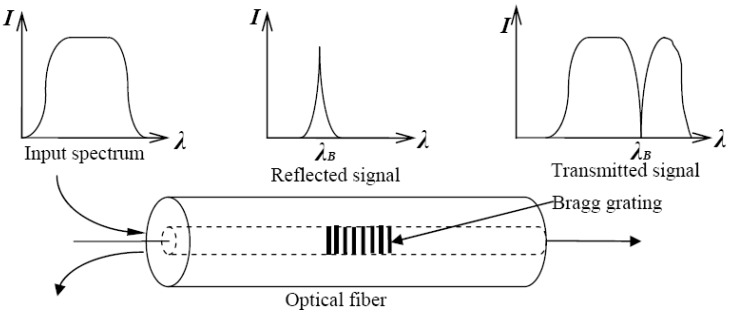
FBG sensor wavelength-encoding operation.

FBG sensors have been regarded as excellent transducers for a wide variety of engineering applications. One of the most commonly-used fiber optic sensors for strain and temperature measurement is the FBG sensor which was developed by Hill *et al.* in 1978 [15].They are immune to electromagnetic interference and they are small enough to be embedded into structures without causing any structural defects. The phase mask method is commonly adopted for fabrication of FBG sensors due to its high performance and simple fabrication process [16]. An FBG is composed of a refractive index “written” by the exposure to an intense UV interference pattern in the core of an optical fiber. If a broadband light is injected into the FBG sensor, it reflects the wavelength corresponding to the spacing of the different gratings, called the Bragg wavelength. The Bragg condition is expressed as the following Equation (1):

(1) $$\lambda_{B}\left(z,\;t\right)=2n_{eff}\left(z,\;t\right)\text{Λ}\left(z,t\right)$$

where $\lambda_{B}\left(z,\;t\right)$ is the Bragg wavelength, $n_{eff}\left(z,\;t\right)$ is the effective refractive index of the core mode, and Λ(z,t) is the grating period of index modulation. The wavelength, which corresponds to the Bragg condition, is reflected at the Bragg grating, and the other wavelengths pass through (see Figure 1). With this approach, the core refractive index of a bare fiber (length of 6 mm) is permanently changed after being exposed to a spatial pattern of ultraviolet light.

The Bragg wavelength $\lambda_{B}\left(z,\;t\right)$ of the FBG sensor will change linearly with the applied temperature ΔT and strain ε. This relationship can be described by Kersey *et al.* [17] as below:

(2) $$\frac{\text{Δ}\lambda_{B}}{\lambda_{B}}=C_{\varepsilon }\varepsilon +C_{T}\text{Δ}T$$

where $\lambda_{B}$ is the original Bragg wavelength under strain free and 0 °C condition, which is the origial condition and the other conditions could be calculated based on it; $\text{Δ}\lambda_{B}$ is the change in the Bragg wavelength due to the variation of strain and temperature; $C_{\varepsilon }$ and $C_{T}$ are the calibration coefficients of strain and temperature. The typical values of $C_{\varepsilon }$ and $C_{T}$ are approximately 0.78 × 10−6$\;{\text{με}}^{-1}$ and 6.7 × 10−6$\;\;{℃}^{-1}$ respectively [18,19]. The above relationship could be expressed more specifically as below:

(3) $$\frac{\text{Δ}\lambda_{B}\left(z,\;t\right)}{\lambda_{B}\left(z,t\right)}=\left(1-p_{e}\right)∙\varepsilon \left(z,t\right)+\left(\alpha +\xi \right)∙\Delta T\left(z,\;t\right)\approx 0.78\varepsilon \left(z,t\right)+6.7\times 10^{-6}\Delta T\left(z,\;t\right)$$

where $p_{e}$ is the elastic optical coefficient; α and ξ are the coefficients of temperature effect; ΔT(z, t) is change of temperature.

### 2.2. Design and Calibration of the FBG Sensing Bar

**Figure 2:**
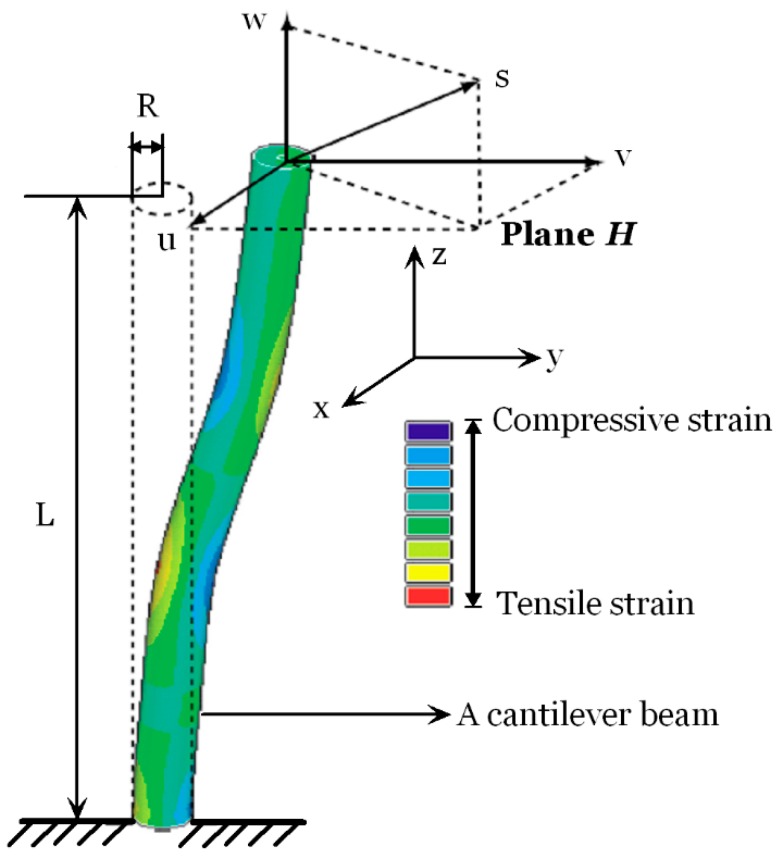
A schematic illustration of a cantilever beam under arbitrary transverse and/or axial loading.

The FBG sensing bars are developed based on the deformations of an elastic axiasymmetric beam as shown in Figure 2. The length and radius of the beam are L and R, respectively. This beam is fixed at one end and subjected to arbitrary transverse and/or axial loading. An imagined plane H passes through the beam perpendicular to z axis. The beam axis on this cross section has deflections u and v in the x and y directions and a tension (or compression) w in the z direction under the effect of load combinations. According to Euler-Bernoulli beam theory [20], the distributions of strain ε(z) is associated with the distributions of normal axial force $F_{N}\left(z\right)$ and the bending moment M(z) along the neutral line of the beam, which can be expressed as below:

(4) $$\begin{array}{c} \varepsilon_{z}=\frac{F_{N}\left(z\right)}{EA}\pm \frac{M_{x}\left(z\right)∙R}{EI_{x}}\pm \frac{M_{y}\left(z\right)∙R}{EI_{y}}\\ =\varepsilon_{A}\left(\text{z}\right)\pm \varepsilon_{Tx}\left(\text{z}\right)\pm \varepsilon_{Ty}\left(\text{z}\right) \end{array}$$

where, $\varepsilon_{A}\left(\text{z}\right)$ is the strains induced by the normal axial force, $\varepsilon_{Tx}\left(\text{z}\right)$ and $\varepsilon_{Ty}\left(\text{z}\right)$ are the strains induced by the transverse loading in the x and y directions, which are associated with the bending moment $M_{x}\left(z\right)$ and $M_{y}\left(z\right)$, respectively. E is the Young’s modulus; A is the cross sectional area; $I_{x}$ and $I_{y}$ represent the moments of inertia with respect to the *x* and *y* axes of the beam, respectively.

The lateral deflections in the *x* and *y* directions and the tensile (or compressive) displacement in the *z* direction can be calculated by integration of strains as the following:

(5) $$\{\begin{array}{c} u=\frac{1}{R}\iint^{\text{​}}\varepsilon_{Tx}\left(\text{z}\right)dzdz\\ v=\frac{1}{R}\iint^{\text{​}}\varepsilon_{Ty}\left(\text{z}\right)dzdz\\ w=\int^{\text{​}}\varepsilon_{A}\left(\text{z}\right)dz \end{array}$$

The displacements of the three directions could be calculated by Equation (5) and the specified boundary conditions. As for the cantilever beam shown in Figure 2, the boundary at the fixed end has the relationships as below:

(6) $$\{\begin{array}{c} u_{0}=v_{0}=w_{0}=0\\ \frac{du}{dz}=0\\ \frac{dv}{dz}=0 \end{array}$$

**Figure 3:**
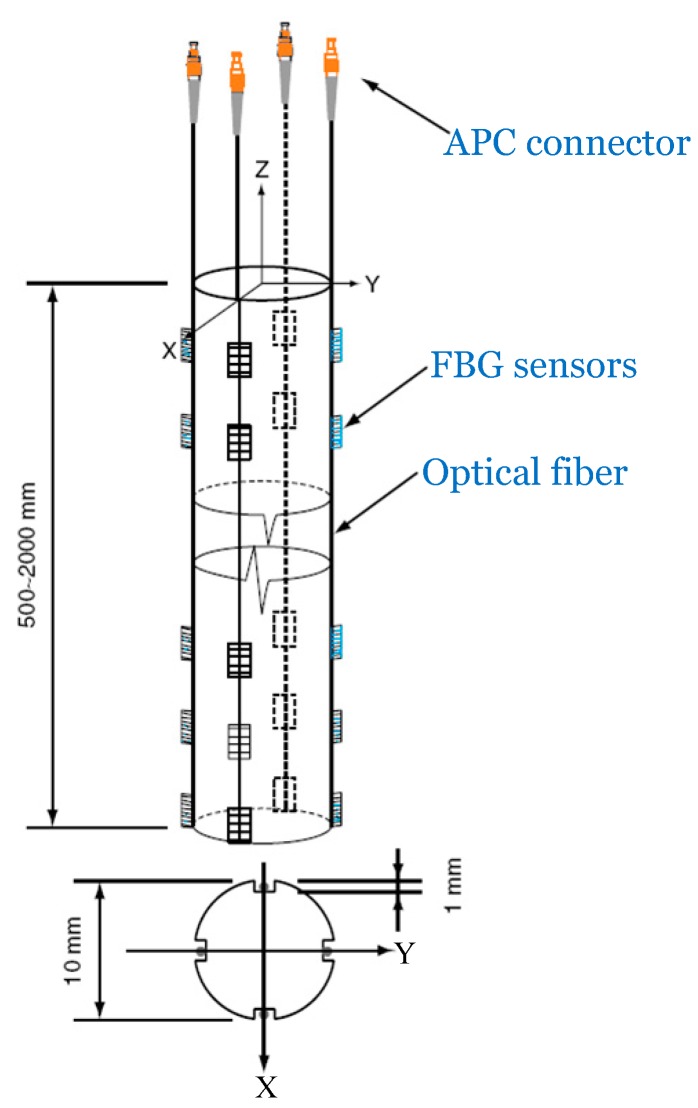
A Design of the FBG sensing bar for 3-D displacement measurement.

Based on the beam theories above, the FBG sensing bar (see Figure 3) is designed and manufactured from a grooved plastic or rubber bar with a diameter of 10 mm. This type of plastic or rubber bar has sufficient elasticity, which could satisfy the cooperation deformation with the surrounding rock mass in the physical model. Four optical fibers are adhered in the grooves and covered with epoxy resin. Each optical fiber contains a series of FBG strain sensors at regular intervals. The length of the bar and the spacing of FBG sensors are changeable according to different experimental conditions.

The FBG sensing bar is designed to be pre-embedded vertically in the process of the construction of a physical model or be inserted into a borehole, working like a cantilever beam under arbitrary axial and/or transverse loading. When the excavations of the cavern group are performed, the FBG sensing bar is subjected to bending and tension (or compression). The multiple FBG sensors measure the strain distributions along the bar perpendicularly. In the plane H in Figure 2, the strains induced by axial and/or transverse loading could be calculated by:

(7) $$\{\begin{array}{c} \varepsilon_{A}^{i}=\frac{1}{4}\left(\varepsilon_{ia}+\varepsilon_{ib}+\varepsilon_{ic}+\varepsilon_{id}\right)\\ \varepsilon_{Tx}^{i}=\frac{1}{2}\left(\varepsilon_{ia}-\varepsilon_{ic}\right)\\ \varepsilon_{Ty}^{i}=\frac{1}{2}\left(\varepsilon_{ib}-\varepsilon_{id}\right) \end{array}$$

where $\varepsilon_{ia}$, $\varepsilon_{ib}$, $\varepsilon_{ic}$, and $\varepsilon_{id}$ are the strains measured by the four FBG sensors on the plane H, respectively. Combining Equations (5) and (7), and performing linear interpolation of strain distributions, the distributions of internal displacements in three dimensions could be obtained.

**Figure 4:**
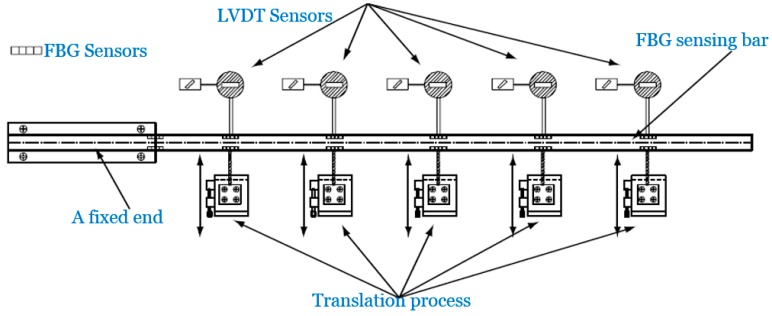
Installment of FBG and LVDT sensors in the deflection test of FBG sensing bar.

**Figure 5:**
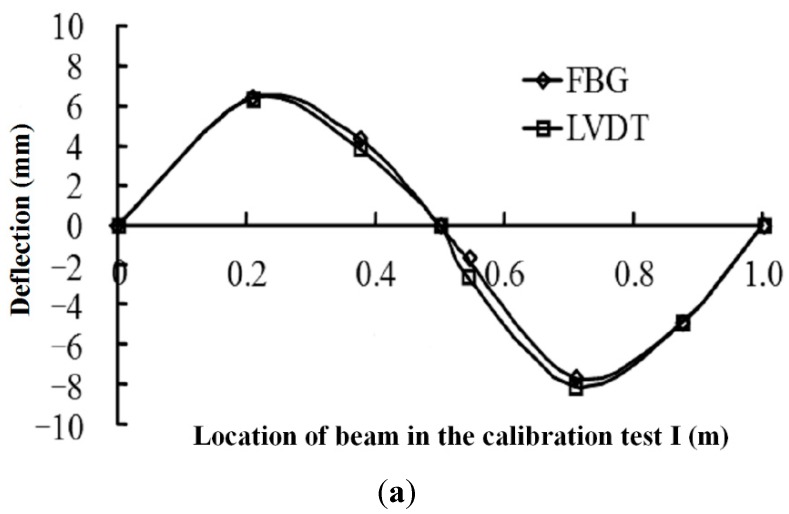

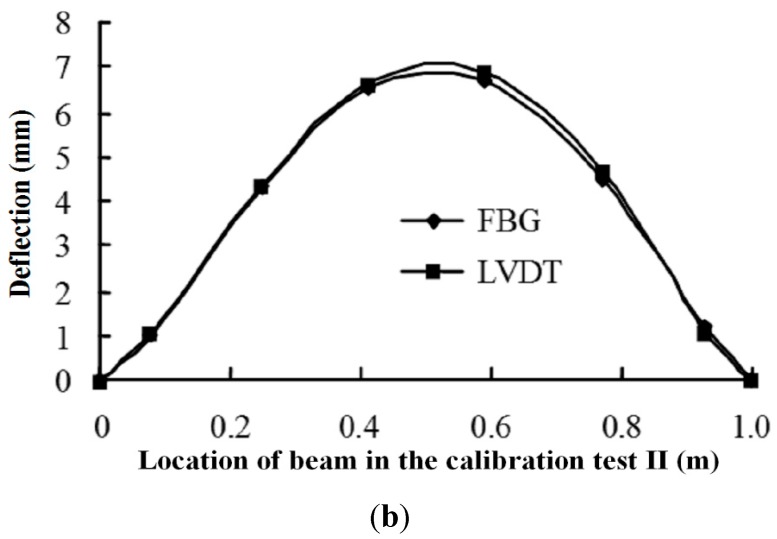
Comparative curves of the deflection values obtained by FBG and LVDT sensors. (**a**) The calibration test of tension; (**b**) The calibration test of deflection.

To verify the effectiveness of the FBG sensing bar, the authors performed a calibration test (tension and deflection) using both the FBG sensing bar and LVDT sensors as shown in Figure 4. During the test, different loads were imposed to the FBG sensing bars. Meanwhile, the deflection values were obtained by using FBG and LVDT sensors. Figure 5 shows the comparative curves of the deflection values obtained by FBG and LVDT sensors.

The calibration tests results show that the FBG sensing bar could easily obtain the applied deflection values along the bar. If the FBG sensing bar is subjected to a lateral displacement at the free end, the relationship between the applied displacement and the maximum strain should be $\frac{D}{\varepsilon_{max}}=\frac{L^{2}}{3R}$. In this deflection calibration test, the resolution of FBG sensing bar for measuring deflection could be calculated as $1^{2}$/($3\times 5\times 10^{-3}$)$\times 1\times 10^{-6}=6.7\times 10^{-5}m=67\;\text{μm}$, and the maximum measuring deflection for the FBG sensing bar could be obtained as $1^{2}$/($3\times 5\times 10^{-3}$)$\;\times 3000\times 10^{-6}=0.2\;\text{m}=200\;\text{mm}$. From the above comparative curves, it is concluded that the deflection measured by the FBG sensors is in good agreement with those obtained by LVDT sensors. Moreover, the FBG sensing bar is an appropriate tool for measuring internal displacement in the physical model test.

## 3. Introduction to the Physical Model Test

### 3.1. Project Description

The Shuangjiangkou Hydropower station is located on the Dadu River in Sichuan Province, China. The river meanders in an entrenched valley with a wall height to 1000 m and slope between 35° and 60°. Below an altitude of 2800 m the valley is a near-symmetrical V-shape. *In situ* stress fields near the underground cavern complex are strongly influenced by the incised terrain, active tectonics, and the high rate of incision and corresponding unloading at the site. *In situ* stresses reach 38 MPa at a depth of approximately 600 m. The rock mass is composed of medium to fine-grained granites with no apparent foliation.

**Figure 6:**
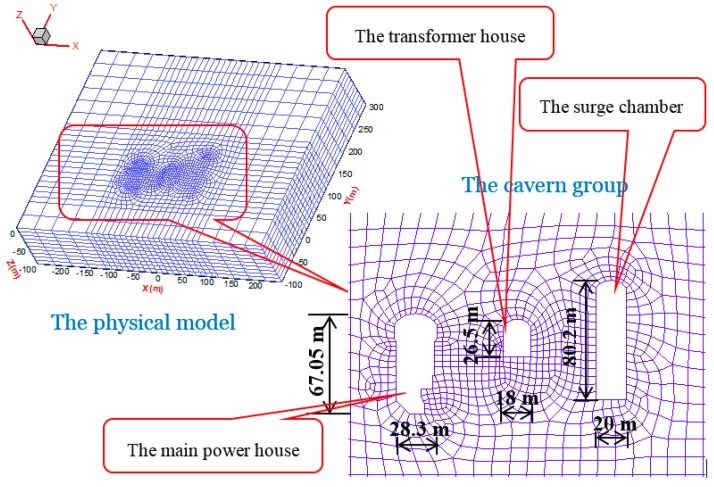
The layout of the physical model with the cavern group.

The underground power-house contains four turbines with a total capacity of 2 GW. The underground cavern group consists of the main powerhouse, the transformer house, and the surge chamber (see Figure 6). The axial direction of the cavern complex is N100W. From observations at other large to medium-sized underground hydropower stations such as Ertan, Xiluodu, Xiaowan, Pubugou, and Jinping hydropower stations, the spacing between the three main caverns was determined from a preliminary stability analysis [1]. The result was to separate the main powerhouse and the transformer house by a 45 m wide pillar, and to separate the transformer house and the surge chamber by 40 m. For the main powerhouse, the transformer house, and the surge chamber, the heights are 67.05 m, 26.5 m, and 80.2 m, respectively. Their spans are 28.3 m, 18 m, and 20 m, respectively.

### 3.2. Three Dimensional Physical Model Test of an Underground Cavern Group

Physical model tests were conducted to investigate the stability of the cavern group, which contains three openings, all subjected to high *in situ* stresses. The scaling ratio between the physical model and the prototype is 1/200.

#### 3.2.1. Steel Structure Frame for the Physical Model Test

**Figure 7:**
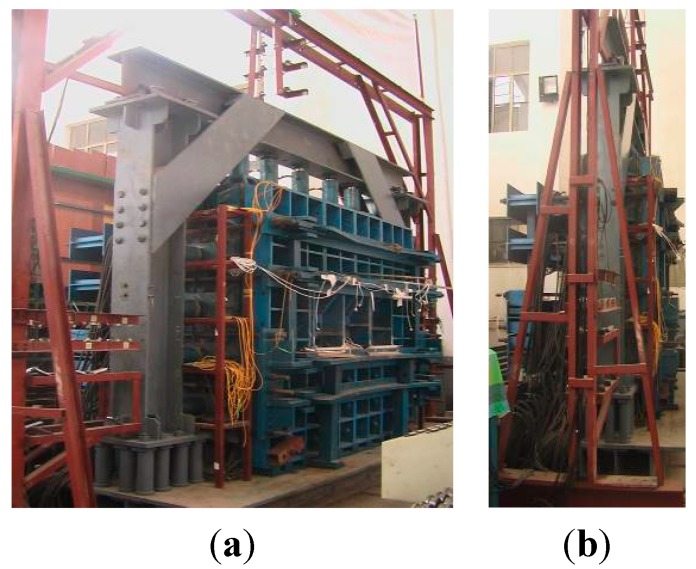
The 3D steel frame structure for the physical model test. (**a**) A front view; (**b**) A side view.

The first step of the physical model test is to design and manufacture a steel structural frame. This steel structure guarantees that the physical model test is in a true three-dimensional state. This structure has technical advantages such as high stiffness, great stability and flexibility of assembly, and easy adjustment of its dimensions. Figure 7 shows the whole true 3D steel frame.

#### 3.2.2. Analogous Material for Physical Model Test

A new type of rock analogy material has been developed in the physical model test, which is made from iron mineral powder, barite powder, quartz powder, and alcoholic solution with rosin. The iron mineral powder, barite powder, and quartz powder are skeletal materials while the alcoholic solution with rosin is a cementing agent. Table 1 shows the physico-mechanical parameters of the rock mass and analogy material.

**Table 1:** The physico-mechanical parameters of the prototype and analogy material.

Type of Material	Density (KN/m^3^)	Young’s Modulus (MPa)	Cohesion (MPa)	Internal Friction Angles (°)	Compressive Strength (MPa)	Poisson’s Ratio
Rock Mass	26.5	3000	2	40.36	80	0.2
Analog Material	26.5	15	0.01	40.36	0.4	0.2

#### 3.2.3. The Installation of FBG Sensing Bar in the Physical Model

**Figure 8:**
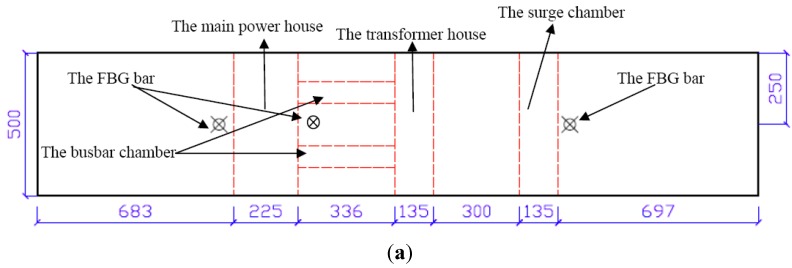

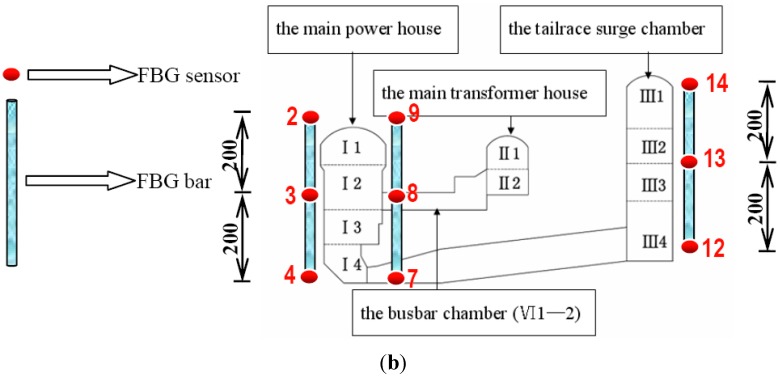
Layout of the FBG sensing bars (Unit: mm). (**a**) A top view; (**b**) A front view.

During the construction of the model, three holes were prepared for installation of FBGs near the openings of the main powerhouse and the surge chamber. The holes were 2000 mm in length and 18 mm in diameter. The center of the hole was 19 mm away from the side wall of the cavern, as shown in Figure 8. The sensing bars were embedded in the model vertically, in order to satisfy the deformation compatibility between the bar and the model. Initially, the ordinary rubber, latex, and silica gel were selected to be the bonding material. After a large number of trial tests, the ordinary silica gel was selected as the bonding material. The sensing bar was inserted into the hole immediately after a reasonable amount of silica gel was injected into the hole as the grouting material. The installation shall be as vertical and smooth as possible so as to avoid axial rotation of the sensing bar. In addition, the direction of the FBGs adhered on the bar shall be perpendicular to the axial direction of the cavern. In this way, the measurement results can truly reflect the deformation of caverns. After 24-hour curing of silica gel, the bonding between sensors and the model was tested to be strong enough, and satisfy the deformation compatibility condition.

#### 3.2.4. Excavations of the Cavern Group and Structural Stability Monitoring of Surrounding Rock Mass

The use of underground caverns in hydropower stations is increasing. However, excavation of such spaces results in a change in stress distribution; the changes alter the mechanical properties of rock mass, such as strength and deformability. In particular, the displacements of critical points at the arch crown and side walls of the caverns are the vital factor to the longevity of the cavern group. If extensive deformation occurs around the caverns, rockburst and failure may occur. Therefore, it is critical to monitor the internal deformation of the underground caverns during excavation in real time.

Due to the space restriction of the model, twenty excavation subsequences in the field were simplified to ten subsequences in the model test (see Figure 8). They are I1, II1, III1, I2, II2, III2, I3, III3, I4, and III4, respectively. The drilling and blasting method was adopted in the *in situ* project, but this method is difficult to be performed in the physical model test. Therefore, the caverns were excavated by special drilling tools with sharp heads. The excavation footage of every substep is 5 cm. The whole excavation subsequences can be divided into 10 circles, and the last two substeps are to excavate the busbar chambers and the other openings, so there are 102 steps in total. During the excavation, the pre-installed FBG sensors took monitoring data automatically. When a circle was completed, the column bolts pre-embedded for bolt holes were pulled out, the rock bolts were placed, and grouting was applied (the column bolts are the same size as the rock bolts, made of slim iron rods). All the pre-installed FBG sensors recorded the data during the excavation. 

## 4. Numerical Simulation of the Physical Model Test and Comparative Analysis with the Monitoring Results Using FBG Sensing Bars

### 4.1. Numerical Simulation of the Physical Model Test

**Figure 9:**
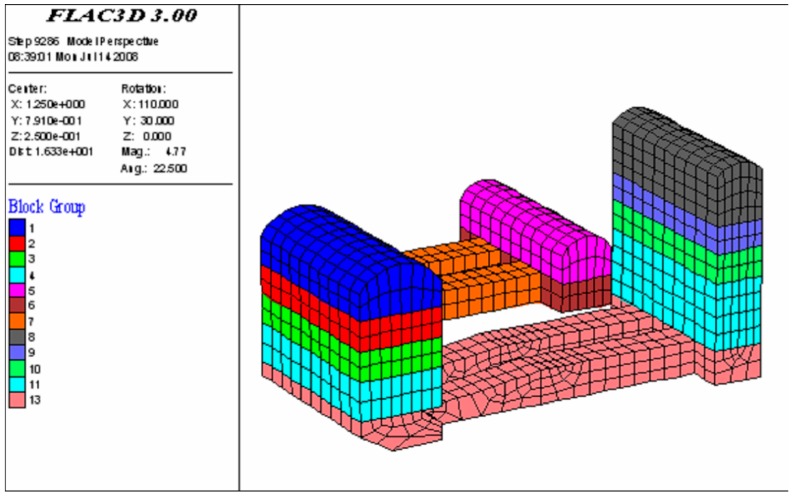
A 3D numerical model of the underground cavern group.

In the numerical simulations, a 3D finite element model, which had the same dimensions as the physical model, was built. The shotcrete layer and the surrounding rock masses were represented by elements with only axial stiffness taken into account. The model was divided into 39,798 nodes and 35,440 elements, as shown in Figure 9. The surrounding rock masses of the underground cavern group were of good integrity, joints did not develop, and seepage of water was not observed in the initial exploration audits. These factors were, therefore, not considered in the numerical simulations. The Drucker-Prager criterion was adopted and the material properties were obtained according to *in situ* physico-mechanical parameters as shown in Table 1. Stability analysis was performed by using the finite-difference method and the FLAC^3D^ code. 

### 4.2. A Comparative Analysis between the Monitoring Results and the Numerical Results

During the excavation process, the FBG monitoring results show that the maximum displacements appear at the middle part of the upstream side wall of the mainpower house and downstream side wall of the surge chamber (*i.e.*, FBG #3 and #13). The maximum displacements are 0.182 mm and 0.257 mm, respectively. It is reasonable that the height of the surge chamber is greater than that of the main powerhouse.

**Figure 10:**
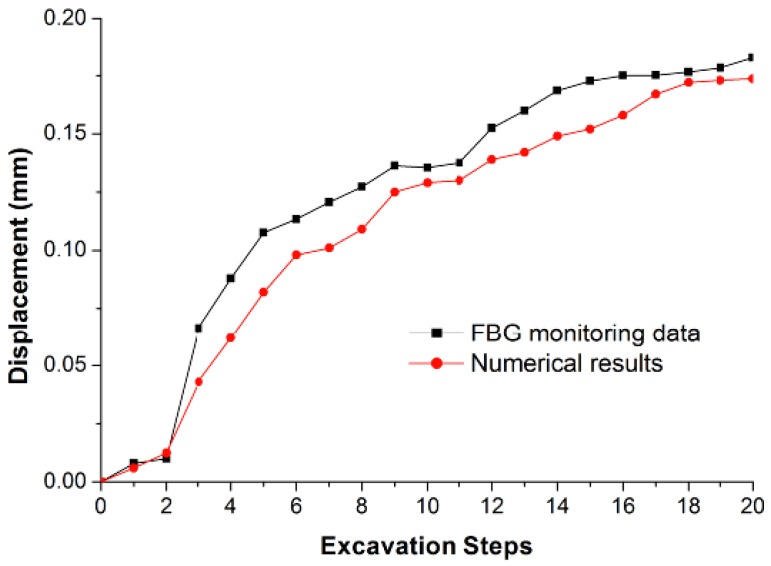
The horizontal displacement curves with the excavation steps obtained by FBG sensing bar and numerical simulation at monitoring point #3.

**Figure 11:**
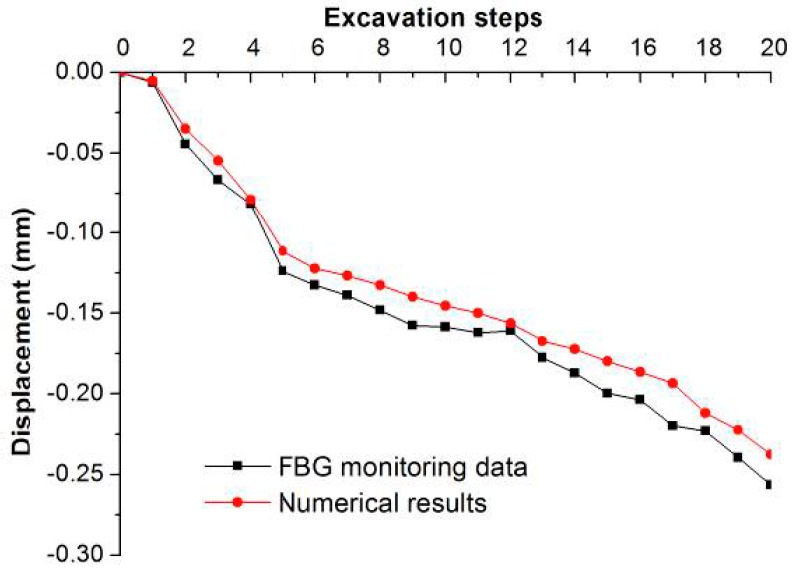
The horizontal displacement curves with the excavation steps obtained by FBG sensing bar and numerical simulation at monitoring point #13.

Figure 10 and Figure 11 show the horizontal displacement curves of FBG #3 and #13. The horizontal axis represents the excavations steps. It is shown that the monitoring results agree well with the numerical results. As the excavations continue, the deformation of each point is increasing, and ultimately tends toward a stable value. 

There was no brittle failure or rockbursts observed during the excavation process. It is concluded that the excavation scheme is feasible and the supporting scheme has great impacts on the reinforcement of the surrounding rock mass.

## 5. Summary and Conclusions

This paper presents the development of a novel FBG sensing bar for monitoring internal displacements of a large-scale physical model test on an underground cavern group. The FBG sensing bar is designed to be embedded in the physical model and measure the internal displacements accurately and automatically. The main conclusions of this work are:

(1) It is feasible to use FBG sensors in the internal displacement monitoring of a large-scale physical model test. The FBG sensor has more advantages than other conventional sensors, such as its small size, high measuring accuracy, high sensitivity, ability to be embedded in the physical model, strong anti-interference ability, wide measurement range, and online continuous detecting ability.
(2) The design and installation measures of FBG sensing bars in the physical model is proved to be successful and worth popularizing.
(3) In the process of the cavern group excavations, the displacements of the surrounding rock mass of the cavern group continue to increase as the excavation go on, but the surrounding rock mass is always in a stable state. In this whole stage, the displacement monitoring results obtained by FBG sensors are in good agreement with those obtained by numerical results.
